# Mechanical performance of narrow dental implants with varying lengths and angled abutments

**DOI:** 10.6026/973206300214589

**Published:** 2025-12-15

**Authors:** Darshana Raut, Gaurav Beohar, Prince Kumar, Mayur Khairnar, Babita Pawar, Nilesh Fatale

**Affiliations:** 1 Department of Prosthodontics and Crown & Bridge, Bhabha College of Dental Sciences, Bhopal, Madhya Pradesh, India; 2 Private Practitioner, Precigem Dental World, Mumbai; 3 Department of Peridontics, Yogita Dental College & Hospital, Khed, Maharashtra, India

**Keywords:** Dental implants, angled abutment, fatigue testing, mechanical properties, narrow diameter

## Abstract

Mechanical performance of narrow-diameter dental implants with varying lengths and abutment angulations remains a critical factor in long-term clinical success. Therefore, it is of interest to evaluate Quickdent implant systems under static and fatigue loading according to ISO 14801:2016E. Group 1 (3.5 mm x 10 mm, 25° abutment) and Group 2 (3.5 mm x 25 mm, 45° abutment) were tested using fifteen assemblies per group. Group 1 showed 418 N static and 182 N fatigue resistance, while Group 2 showed 454 N static and 160 N fatigue resistance with distinct failure modes. Thus, we show that longer implants offer higher static strength, whereas shorter ones perform better under fatigue conditions.

## Background:

Dental implants have revolutionized restorative dentistry, providing a predictable solution for tooth replacement. The success of implant-supported prostheses depends on various factors, including implant design, surface characteristics, and biomechanical compatibility [1, 2]. In clinical practice, anatomical limitations often necessitate the use of angled abutments to achieve optimal prosthetic positioning and esthetic outcomes [3]. However, the introduction of angles in the implant-abutment complex creates potential stress concentration areas that may compromise the long-term mechanical integrity of the system [4]. Narrow diameter implants (typically <3.75 mm) have gained popularity for cases with limited bone width, offering a less invasive alternative to bone augmentation procedures [5]. Despite their clinical advantages, narrow diameter implants may be more susceptible to mechanical complications due to reduced material volume and potentially altered stress distribution patterns [6]. The mechanical performance of these implants under functional loading conditions remains a critical consideration for clinical success [7]. Previous studies have investigated the mechanical behavior of dental implants under various loading conditions [8, 9]. However, limited research has directly compared the performance of different implant lengths with corresponding angled abutments under standardized testing protocols [10]. The ISO 14801:2016E standard provides a methodology for fatigue testing of endosseous dental implants, allowing for comparative evaluation across different implant systems [11]. Recent studies have highlighted the importance of fatigue testing in predicting the long-term performance of dental implant systems [12, 13]. Fatigue failure, which can occur at loads significantly below the ultimate strength of materials, is particularly relevant for dental implants that undergo cyclic loading during masticatory function [14]. The relationship between implant length, abutment angle, and fatigue resistance remains incompletely understood [15]. This research gap is particularly significant given the increasing use of narrow diameter implants with angled abutments in clinical practice [16]. Understanding the mechanical behavior of these systems under both static and fatigue loading conditions is essential for optimizing treatment planning and improving long-term clinical outcomes [17]. Therefore, it is of interest to evaluate and compare the mechanical performance of narrow diameter dental implants with different lengths and corresponding angled abutments under static and fatigue loading conditions, according to ISO 14801:2016E standard.

## Materials and Methods:

## Study design:

A comparative experimental study was conducted to evaluate the mechanical performance of two different narrow diameter dental implant systems under static and fatigue loading conditions. The study protocol followed the ISO 14801:2016E standard for fatigue testing of endosseous dental implants. All tests were performed in an independent testing laboratory specializing in biomechanical evaluation of dental materials.

## Sample size and groups:

Fifteen implant assemblies were tested for each group, providing a total sample size of thirty implants. Group 1 consisted of Quickdent Standard Titanium Angular Abutment 25°, 4mm IH on 3.6mm Quickdent Korticale TPR with a length of 10mm. Group 1 samples included shortest implants and abutment with maximum angle and highest collar. Group 2 comprised Quickdent Standard Multi-unit Angular Hex-45-4-M1.4 on Quickdent Korticale TPR XL Hex implants with dimensions of 3.5 x 25mm. Group 2 samples included highest length implants and Multi-unit Angular abutment with maximum angle and highest collar. All implants and abutments were provided by the manufacturer (Quickdent Dental Implant System) and were from the same production lots to ensure consistency.

## Inclusion and exclusion criteria:

All implants and abutments were inspected prior to testing to ensure they were free from visible defects. Components with manufacturing irregularities, surface damage, or dimensional inconsistencies were excluded from the study. Only components from the specified lots were included to maintain material consistency across samples.

## Testing equipment:

The following equipment was used for testing:

[1] Material Test System (MTS Model 820 for Group 1 and MTS Model 810 for Group 2) with a 250 lb. capacity load range for static tests

[2] Krouse Testing Machine Co. axial fatigue machine designed for low loads for fatigue tests

[3] Strain gage load cells with DATAQ DI-2008 DAQ and dynamic load readout

[4] Tohnichi 60Ncm Torque Gage, Model 6BTG-N, for abutment tightening

All testing equipment was calibrated according to manufacturer specifications and traceable to ANSI Z540-1-1994 and ISO 10012-1 standards.

## Test methods:

For Group 1, the abutments were installed with a torque of 30Ncm. The implants were potted into brass sleeves (modulus of elasticity of 103 GPa) 3.0mm below the bone level datum (top of implant), representing a 3mm bone recession. For Group 2, the abutments and copings were installed with torques of 25Ncm and 15Ncm, respectively. The implants were potted into brass sleeves 3.0mm below the bone level datum (4mm from top of implant), also representing a 3mm bone recession.

## Sample preparation:

## Static testing:

A test fixture was used to hold the implant/abutment system at a specific angle to simulate uncorrected angulation. For Group 1, the system was held at a 35° angle (25° angled abutment under-corrected by 10 degrees). For Group 2, the system was held at a 55° angle (45° angled abutment under-corrected by 10 degrees). A spherical end cap was designed such that the center of the contact point was 11mm above the holding line of the implant measured along the axis of the implant, as specified in ISO 14801:2016E. Test loads were applied to the hemispherical tip with a hardened stainless steel rod greater than 50mm in length, by point contact at the machine interface. This allowed unconstrained motion in the transverse direction without reducing the magnitude of the applied bending moment. The moment arm length was calculated using the abutment drawing dimensions and determined to be 2.10mm for Group 1 and 9.77mm for Group 2. Static tests were conducted at room temperature air (75±5°F) with a crosshead speed of 1.27mm/min (0.05 in/min). The maximum loads prior to failure were recorded, and load versus deflection curves were generated.

## Fatigue testing:

Fatigue testing was conducted using the same test fixture as static testing. The testing particulars were as follows:

[1] Minimum fatigue load was 10% of maximum fatigue load

[2] Test speed was 15 cycles/second

[3] Tests were conducted at room temperature in air

[4] Krouse Testing Machine Co. axial fatigue machine designed for low loads was used

Fatigue tests were performed at various load levels to establish the fatigue curve, with particular attention to the 5,000,000 cycle run-out load. Samples that did not fail after 5,000,000 cycles were considered run-out specimens.

## Failure analysis:

All failed specimens were examined under magnification to identify failure modes. For static tests, failure modes included bell mouth yielding of the implant collar and implant crack below the pot line. For fatigue tests, failure modes included implant crack below or above the pot line, depending on the group.

## Statistical analysis:

Descriptive statistics including mean and standard deviation were calculated for static and fatigue test results. The data were analyzed to determine the average ultimate static load, fatigue run-out load, and corresponding bending moments. No inferential statistics were performed due to the descriptive nature of the study.

## Results:

Group 1 (3.5mm diameter, 10mm length implants with 25° angled abutments) demonstrated an average ultimate static load of 94.1 lbf (418N). The failure modes observed were bell mouth yielding of the implant collar (33.3%) and implant crack below the pot line (66.7%). Group 2 (3.5mm diameter, 25mm length implants with 45° multi-unit abutments) exhibited an average ultimate static load of 102.0 lbf (454N). The failure modes were implant crack above the pot line (33.3%) and yielding of the implant collar (66.7%). Table 1 (see PDF) summarizes the static testing results for both groups, including maximum loads and failure modes. For Group 1, the fatigue 5,000,000 cycle run-out load was 41 lbf (182N) with a corresponding bending moment of 38.3 Ncm. Three implant systems reached the run-out condition without failure. The primary failure mode observed during fatigue testing was implant crack below the pot line. For Group 2, the fatigue 5,000,000 cycle run-out load was 36.0 lbf (160N) with a corresponding bending moment of 156.4 Ncm. Four implant systems reached the run-out condition. The failure modes during fatigue testing were implant crack at the pot line and implant crack above the pot line. Table 2 (see PDF) presents the fatigue testing results for both groups, including maximum loads, bending moments, and cycles to failure. Table 3 (see PDF) provides a comparative summary of the key findings between the two implant systems, including static load capacity, fatigue run-out load, bending moments, and predominant failure modes. The load versus deflection curves for static tests and fatigue curves for both groups were generated, demonstrating the mechanical behavior of each implant system under the respective loading conditions (Figure 1 - see PDF).

## Discussion:

The present study evaluated and compared the mechanical performance of two narrow diameter dental implant systems with different lengths and angled abutments under static and fatigue loading conditions. The results provide valuable insights into the biomechanical behavior of these systems, which is essential for clinical decision-making and treatment planning. Group 2, featuring longer implants (25 mm) with 45° multi-unit abutments, demonstrated a higher average ultimate static load (454 N) compared to Group 1 with shorter implants (10 mm) and 25° angled abutments (418 N). This finding is consistent with previous research suggesting that longer implants provide increased resistance to static loads due to enhanced bone-implant contact and improved load distribution [18]. The additional implant length in Group 2 likely contributed to greater stability and resistance to deformation under static loading conditions. However, it is important to note that the difference in static load capacity between the two groups was relatively modest (approximately 8.6%), despite the significant difference in implant length (15 mm). This suggests that factors beyond implant length, such as abutment design and connection geometry, also play crucial roles in determining the static load resistance of implant systems [19]. The multi-unit abutment design in Group 2 may have introduced additional stress concentration areas that partially offset the benefits of increased implant length. Interestingly, Group 1 demonstrated a higher fatigue run-out load (182 N) compared to Group 2 (160 N) at 5 million cycles. This finding contradicts the static test results and suggests that shorter implants may offer advantages in fatigue resistance under the tested conditions. The enhanced fatigue performance of Group 1 may be attributed to several factors, including the lower test angle (35° vs. 55°), which resulted in reduced bending moments, and the simpler abutment design with fewer potential stress concentration areas [20]. The bending moment at the run-out load was significantly lower for Group 1 (38.3 Ncm) compared to Group 2 (156.4 Ncm), primarily due to the difference in moment arm length (2.10 mm vs. 9.77 mm). This substantial difference in bending moment may explain the superior fatigue performance of Group 1 despite its shorter implant length. These findings highlight the importance of considering both implant length and abutment design when evaluating the fatigue performance of implant systems [21]. The analysis of failure modes revealed distinct patterns between the two groups. In Group 1, the predominant static failure mode was implant crack below the pot line (66.7%), while in Group 2, yielding of the implant collar was more common (66.7%). These differences suggest that the stress distribution patterns vary significantly between the two systems, likely due to differences in implant length, abutment angle, and connection design [22]. During fatigue testing, Group 1 primarily exhibited implant cracks below the pot line, while Group 2 showed both implant cracks at and above the pot line. The variation in failure locations indicates different stress concentration areas in the two systems, which may have implications for clinical performance and long-term prognosis [23]. The consistent failure patterns observed within each group suggest that these are characteristic failure modes for the respective implant-abutment configurations. The findings of this study have several important clinical implications. The higher static load capacity of Group 2 suggests that longer implants may be preferable in situations where high immediate loads are anticipated, such as in immediate loading protocols or in patients with strong masticatory forces [24]. However, the superior fatigue performance of Group 1 indicates that shorter implants with appropriately designed abutments may offer advantages for long-term functional loading, particularly in cases with significant angulation corrections [25]. The differences in failure modes between the two systems also have clinical relevance. Implant cracks below the pot line (predominant in Group 1) may be more challenging to detect clinically and could potentially lead to catastrophic failures. In contrast, yielding of the implant collar (predominant in Group 2) might be more readily detectable during routine follow-up examinations, allowing for earlier intervention [26]. Several limitations should be considered when interpreting the results of this study. First, the tests were conducted in vitro under controlled laboratory conditions, which may not fully replicate the complex oral environment with factors such as saliva, pH variations, and dynamic thermal changes [27]. Second, the implants were embedded in brass sleeves rather than bone, which may have influenced the stress distribution patterns and failure modes [28]. Additionally, the study focused on specific implant-abutment configurations from a single manufacturer, which may limit the generalizability of the findings to other implant systems [29]. The sample size, while adequate for descriptive analysis, was relatively small and may not capture the full range of potential variations in mechanical performance [30]. Future studies should expand on these findings by investigating a broader range of implant lengths, diameters, and abutment angles to establish more comprehensive performance profiles [31]. Additionally, research incorporating more complex loading conditions, including thermomechanical cycling and multi-axial loading, would provide further insights into the clinical performance of these systems [32]. Long-term clinical studies are also needed to validate the in vitro findings and establish correlations between laboratory testing results and clinical outcomes [33]. Such studies would help clinicians make more informed decisions when selecting implant systems for specific clinical scenarios [34].

## Conclusion:

Both narrow-diameter implant systems exhibited satisfactory mechanical integrity under static and fatigue loading. Longer implants provided enhanced static strength, whereas shorter implants showed superior fatigue endurance. Clinically, implant selection should balance these characteristics based on prosthetic angulation and anticipated functional loading demands.
